# Trained Immunity Carried by Non-immune Cells

**DOI:** 10.3389/fmicb.2018.03225

**Published:** 2019-01-14

**Authors:** Attoumani Hamada, Cédric Torre, Michel Drancourt, Eric Ghigo

**Affiliations:** IRD, MEPHI, Institut Hospitalier Universitaire Méditerranée Infection, Aix-Marseille University, Marseille, France

**Keywords:** trained immunity, immunomodulatory, non-immune cells, stem cells, lifespan

## Abstract

“Trained immunity” is a term proposed by Netea to describe the ability of an organism to develop an exacerbated immunological response to protect against a second infection independent of the adaptative immunity. This immunological memory can last from 1 week to several months and is only described in innate immune cells such as monocytes, macrophages, and natural killer cells. Paradoxically, the lifespan of these cells in the blood is shorter than the duration of trained immunity. This observation suggested that trained immunity could be carried by long lifespan cells such as stem cells and non-immune cells like fibroblasts. It is now evident that in addition to performing their putative function in the development and maintenance of tissue homeostasis, non-immune cells also play an important role in the response to pathogens by producing anti-microbial factors, with long-term inflammation suggesting that non-immune cells can be trained to confer long-lasting immunological memory. This review provides a summary of the current relevant knowledge about the cells which possess immunological memory and discusses the possibility that non-immune cells may carry immunological memory and mechanisms that might be involved.

## Introduction

Works regarding T cell and B cell-independent immune memory date back over half a century (Box 1). A first report by Mackaness (1968) showed that mice vaccinated against tuberculosis with the vaccine bacillus Calmette-Guérin (BCG) were effectively protected against a second infection by mycobacteria. Further, it was shown that trained immunity lasted for weeks to months (Cassone, 2018) and other infectious agents such as *Salmonella typhimurium, Listeria monocytogenes, Staphylococcus aureus, Candida albicans*, and *Schistosoma mansoni* (Blanden et al., 1969). These results were confirmed by Tribouley et al. showing the protective effect of BCG on athymic mice against *Schistosoma mansoni* (Tribouley et al., 1978). In the 80–90s, Bistoni and his colleagues showed that mice infected with attenuated *Candida albicans* exhibited protection against a lethal dose of *Candida albicans* and other pathogens such as *Staphylococcus aureus* (Bistoni et al., 1986). This protection was independent of acquired adaptative immune cells (Box 2) but depended on the innate immune cells as macrophages and a higher production of pro-inflammatory cytokines including interleukin (IL)-1, granulocyte macrophage colony stimulating factor (GM-CSF), tumor necrosis factor (TNF)-α and interferon (IFN)-γ (Bistoni et al., 1986; Vecchiarelli et al., 1988). Then, several studies have shown that in the same way as monocytes, NK-cells exhibit immunological memory. O'Leary et al. showed that a hapten (small molecule triggering an immune response) (Erkes and Selvan, 2014) induced contact hypersensitivity in T and B cell-deficient mice during the second contact with same hapten (O'Leary et al., 2006). This activity was shown to be carried by a liver subpopulation of NK cells (Ly49C-I+) (O'Leary et al., 2006). Perforin and granzyme were the factors related to the defense mechanisms of NK-cells (Salcedo et al., 1993). The production of these effectors are controlled by promotor of gene, regulator sequence (enhancer–silencer) and transcription factors such as lymphotoxin α (*LTA*), tumor necrosis factor α (*TNFA*), lymphotoxin β (*LTB*) and interferon-γ (*IFNG*) genes which may undergo epigenetic modifications (Cichocki et al., 2013; Wiencke et al., 2016). It needs to be investigated how the mechanisms of production of perforin and granzyme may be supported by an epigenetic modification. As for mast cells, there is no published study related to immunological memory. Nevertheless, Monticelli hypothesized that mast cells, like the other innate immune cells, could adopt a memory phenotype (Monticelli and Leoni, 2017). Indeed, mast cells play an important role in the first line defense and possess a longer life than monocyte/macrophage and NK-cells. Also, mast cell biology can be regulated by epigenetic modifications as described in monocytes/macrophages and NK-cells. They play a critical role in the establishment and maintenance of mast cell identity, expansion, differentiation and regulation of mast cell response to a danger signal (Montagner et al., 2016; Monticelli and Leoni, 2017). A recent study reports that the DNMT3A DNA methyltransferase is important to modulate mast cell responses to chronic stimuli (Leoni et al., 2017). Therefore, mast cells could support an immunological memory, which remains to be investigated. At last, dendritic cells (DCs) possess the immunological arsenal against bacteria; have a longer lifespan than monocytes and epigenetic mechanisms to support trained immunity. However, there is no study reporting trained immunity for DCs.

Box 1 **Innate immunity.**

Innate immune system is the second line of defense against microbial infection in vertebrates and non-vertebrates. It helps containing most infectious agents behind the first line anatomical and physiological defenses (Turvey and Broide, 2010). The innate immune system comprises hematopoietic cells including mast cells, macrophages, dendritic cells, neutrophils, and eosinophils derived from myeloid lineage and natural killer (NK) cells. When a microorganism succeeds in crossing the anatomical and physiological barriers, the innate immune system takes over to efficiently remove it. Innate immunity develops in several steps including recognition of the microorganism via surface pattern recognition receptors shared by all the innate immune cells, binding to common pathogen-associated molecular patterns (PAMPs), uptake of the microorganism and induction of a production of inflammatory cytokines, chemokines, and chemotractant molecules to recrute additional immune cells. The processus of microbial clearance continues and immature dendritic cells which uptook the microorganism migrate to lymph nodes to initiate adaptative immunity (Zak and Aderem, 2009; Turvey and Broide, 2010).

Box 2 **Adaptative immunity.**

Adaptative immunity is the third line of defense composed by hematopeitic T cells and B cells derived from the *lymphoid lineage*. Dendritic cells recognize pathogen, uptake it, process microbial antigens and migrate in the secondary lymphoid organs to encounter naive T cells. Dendritic cells present the antigens by the human major histocompatibility (HMC)-II molecules to naive T cells (Th0). T-lymphocytes specific to the antigen are activated, leading to their clonal expansion and differentiation into effector T cells such as Th1, Th2, and Th17 cells. Functionally, effector T cells prime different types of immunity such as cellular immunity, humoral immunity and tolerance immunity and secrete pro-inflammatory cytokines such as IFN-γ, IL-2, IL-4, IL-6, IL-17, and TNF-α to activate other immune cells, contributing to pathogen clearance. Some long half-life T cells become memory T cell and have the capability to quickly respond to subsequent exposure to the same pathogen (specific-protection; Clark and Kupper, 2005; Pennock et al., 2013).

In Kleinnijenhuis et al. (2012) highlighted the mechanism involved in the immune protection previously observed by Bistoni and others. Indeed, these authors showed that reprogramming cells and inflammatory response conferred to monocytes/macrophages were associated with epigenetic modification mechanisms. Briefly, naive cells have a compacted DNA rendering it inaccessible to promoters/enhancers. After infection, the DNA decondensates making it accessible to the promoters/enhancers that could be mono-methylated and mono-acetylated in a way such that the DNA will be transcribed, allowing for the production of molecules involved in pathogen elimination (Mehta and Jeffrey, 2015). Once the infection is resolved, the acetylation is lost but methylation of lysine 4 of histone 3 (H3K4 me) persists and keeps the promoters active so that during the second stimulation the DNA is rapidly transcribed allowing great production of genes involved in immunity (Ostuni et al., 2013; Quintin et al., 2014; Saeed et al., 2014). Metabolic induction is additional mechanisms underlying trained immunity (Bekkering et al., 2018).

Following pioneering work by Netea, the concept of “trained immunity or innate immune memory” has been proposed (Netea et al., 2011) (Box 3).

Box 3 **Trained immunity.**

Trained immunity is a new concept designing the adaptative properties carried by innate immune cells such as macrophages and NK-cells. Innate immune cells including monocytes/macrophages and NK-cells are primed by recognition of PAMP such as lipopolysaccharides, bacterial DNA, mannans that bind to Toll-like receptors (TLRs), and Nod-like Receptors (NLRs) (Janeway and Medzhitov, 2002; Kleinnijenhuis et al., 2012). Priming induces a high protective inflammatory response via the release of cytokines such as IFN-γ conferring a protection to a secondary presentation of PAMPs carried by the same or a different pathogen than the one, which primed trained immunity (cross-protection). Epigenetic modifications and immunometabolism are underlying mechanisms for training immune cells to act efficiently during a second infection (Netea et al., 2015).

Recently, Cheng et al. showed a new mechanism to support “trained immunity” focusing especially on the metabolism of cells such as glycolysis. For example they showed that the cholesterol synthesis pathway was highly induced in β-glucan-trained macrophages (Cheng et al., 2014). The interplay between metabolite production and trained immunity has been recently shown (Arts et al., 2016a). Cholesterol synthesis pathway requires an intermediate metabolite called mevalonate; which induces a trained immunity profile by promoting the expression of a set of genes required in β-glucan-trained immunity phenotype like fumarate (Arts et al., 2016a; Bekkering et al., 2018). Trained immunity induces an aerobic glycolysis associated with an augmentation of glucose consumption, lactate production and elevated intracellular ratio of nicotinamide adenine dinucleotide (NAD+/NADH) (Cheng et al., 2014).

**Figure 1:**
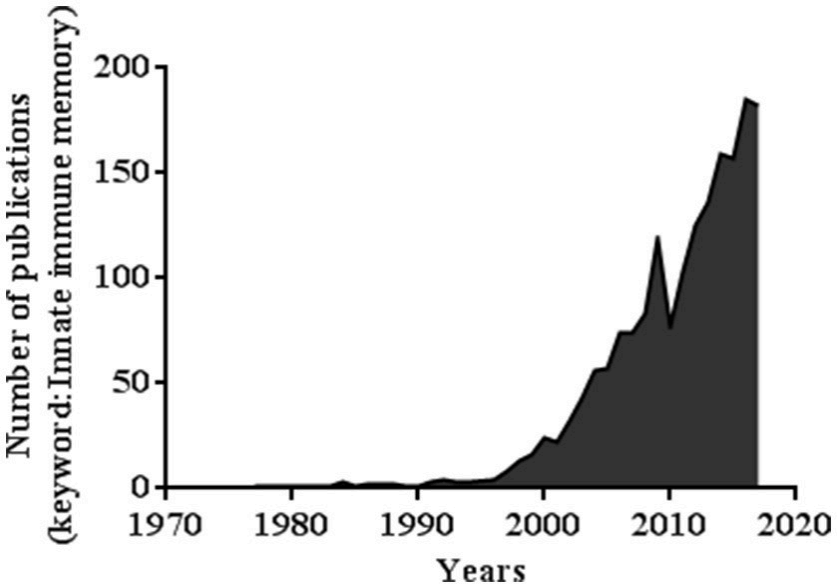
Number of publications related to the keywords “Innate immune memory” (PubMed database).

Trained immunity draws more and more attention and interest. A simple investigation of the number of publications based on the key words “innate immune memory” between 1969 and 2018 shows a sharp increase in the number of papers, reaching 142 papers in July 2018 (Figure 1).

Despite new knowledge, fundamental questions need to be addressed about the paradoxe between the duration of trained immunity and the short lifespan of innate immune cells (Table 1). Until now all of the studies were performed in monocytes/macrophages and NK cells whereas trained immunity can last for 1 year and more.

**Table 1:** Characteristics of the trained immunity compared to cell involved.

	**Types of cells**	**Life Span**	**Priming agents**	**Mechanism underlying**	**Consequences**	**References**
**Innate immune cells**	Monocytes Macrophages	7 days	BCG	Epigenetics modifications: (H3K4me3 persistency), Increased H3K4 trimethylation monocytes after BCG Vaccination and training BCG vaccination dependent to NOD2 and Rip2	BCG induces immunological memory protection through reprograming cells, inflammatory response, increase of cytokine production (IFN-γ, TNF, and IL-1β)	Kleinnijenhuis et al., 2012
			*Candida albicans*	Epigenetic modifications: increase of H3K4me3.	Protection against reinfection induced by Candida albicans. Pro-inflammatory protective response TNF-alpha, IL-6, and IL-18.	Quintin et al., 2012
	NK-cells	6 Months	Hapten-induced contact	Trained immunity carried by NK-cells (Ly49)	Inflammatory memory induced against hapten 2,4-dinitro-1-fluorobenzene [DNFB] or oxazolone	O'Leary et al., 2006
			MCMV	Reprograming NK-cell with pro-inflammatory cytokines signals operating through IL-12 and STAT4	MCMV-specific NK cell clonal expansion as well as memory NK cell formation: protection against MCMV Infection	Sun et al., 2012
**Non-immune cells**	Hematopoeitic Cells	Indefinite lifespan	BCG	BCG induce epigenetic modification for three histone marks (H3K4me1, H3K4me3, H3K27AC).	Expansion of HSC, Myelopoeisis, BCG train HSCs to generate trained monocytes/macrophages, high production of cytokine essential for protective antimycobacterial	Kaufmann et al., 2018
			β-glucan	Immunometabolic pathways β-glucan induce an increase of glycolysis in train HSCs	Expansion of HSPCs, IL-1b GM-CSF	Mitroulis et al., 2018
	Mesenchymal Stem Cells	Indefinite lifespan	LPS	Epigenetic mechanism: miRNAs (miR146a, miR150, and miR155, along with the modicifation of DNA by 6hydroxymethylcytosine (5hmC)	Increased expression of pro-inflammatory cytokine IL-6, IL-8	Liu et al., 2016
	Epithelial Stem Cells (EpSCs)	Indefinite lifespan	Imiquimod (IMQ)-induced model of skin inflammation	Epigenetic modifications: induced epithelial stem cells maintains chromosomal accessibility of both epidermal and inflammation genes after the first stimulus. In the second stimulus genes were transcribed rapidly.	Inflammatory memory carried by non-immune cell (EpSCs) of the skin. Accelerating wound repair in induced mice 2.5 times faster than naive.	Naik et al., 2017

Consequently, trained immunity is more and more investigated in non-immune cells such as stem cells, which possess immune characteristics (expression of TLRs, inflammatory response, production of antimicrobial peptides) with long life span.

### Epigenetic Mechanism

Term composed by “epi” meaning “above” in Greek and “genetic” relating to genes. Basically, it is the set of chemical modifications occurring in the DNA and consequently modulating the expression of genes (Box 4). The mechanism does not affect the sequence of the DNA but is transmissible to the offspring. Epigenetic modifications include DNA methylation, histone methylation and acetylation (Saeed et al., 2014; Hoeksema and de Winther, 2016). In general, DNA methylation is an epigenetic mechanism which involves the addition of a methyl-CH3 group on carbon predominantly to the CpG dinucleotides of the cytosine residues of DNA 5-methylcytosine (5 mC). This process involving three DNA methyltransferases (DNMT1, DNMT3A, and DNMT3B) is active in the regulation and maintenance of gene expression (Jaenisch and Bird, 2003). NK-cell memory trained by BCG is associated with DNA methylation (Sun et al., 2012; Schlums et al., 2015).

Box 4 **Molecular and metabolic mechanims involved in trained immunity.**

Epigenetic events are part of the mechanisms controlling the way innate immune cells maintain the immune response.

### Immunometabolic Mechanism

Contraction of “immune” and “metabolic,” originally proposed to explain both the cellular metabolism of innate immune cells and the role of immune cells playing in metabolic diseases and/or organ metabolism in global. The first study on immunometabolic mechanism concerned the relation between immunity and metabolic diseases, diabetes, and obesity (Ferrante, 2013). Recent studies showed that metabolic mechanisms are also highlighted in trained immunity (Arts et al., 2016b; Hotamisligil, 2017). In monocytes trained by β-glucan, transcriptional and epigenetic analysis revealed an increase in the promoters of genes encoding enzymes involved in glycolysis (hexokinase and pyruvate kinase) and its master regulator mTOR (mammalian target of rapamycin) (Cheng et al., 2014).

## Trained Immunity of Non-Immune Cells

### Stem Cells

#### Mesenchymal Stromal/Stem Cells

Mesenchmal stem cells possess immune properties such as immunosuppressive phenotype, inflammatory phenotype, anti-bacterial characteristics and are equipped with Pattern Recognition Receptors (*PRRs*), including Toll-Like Receptors (TLRs) (Pevsner-Fischer et al., 2007; Liotta et al., 2008; Coffelt et al., 2009; Krasnodembskaya et al., 2010; Machado et al., 2013). In 2006, Guang-yang Liu and his colleagues showed that adipose mesenchymal stem cells exhibit short-term memory when exposed a second time to bacterial ligand LPS or danger signal TNF-α. Precisely, mesenchymal stem cells primed with LPS or TNF have the ability to produce more intensely pro-inflammatory cytokines IL-8, MCP-1, and IL-6 to the same stimulus upon a second encounter. Additionally, they showed that primed mesenchymal stem cells exhibited a better therapeutic effect on diabetic rat model than unprimed MSCs (Liu et al., 2016). Moreover, like in monocytes, this trained immunity is governed by epigenetic mechanisms. The authors showed the involvement of a set of microRNAs (miR146a, miR150, and miR155) and a modification of by 5-hydroxymethylcytosine (5 hmC) (Liu et al., 2016). This study was the first one to demonstrate that trained immunity could be carried by non-professional immune cells, Nevertheless, it concerns a short-term memory only corresponding just to 7 days whereas the trained immunity can last from 1 week to months and several years (Nguipdop-Djomo et al., 2016). Recently, it has been shown that the planarian *Schmidtea mediterranea* is exhibiting trained immunity function (Torre et al., 2017). Planarian previously infected with *Staphylococcus aureus* developed an improved immune response with increased bacterial clearance during a second infection. This immunological memory is due to stem cells that support cell replacement during homeostasis and regeneration of any missing tissue (Eisenhoffer et al., 2008; Kaufmann et al., 2018). RNAi-based experiments revealed the signaling Smed-PGRP-2/Smed-setd8-1 methyltransferases as key factors in instructed neoblastes (planarian stem cells) (Torre et al., 2017). This study provided additional information to explain the capacity of BCG to induce a long immune memory (30 days minimum) and to persist for months to years whereas the lifespan of many innate immune cells in circulation is limited on the order of hours or days (Sun et al., 2011; Kleinnijenhuis et al., 2012; Nayar et al., 2015). Eventually, Kauffman et al. showed that priming HSCs with intravenous BCG vaccine reaching the bone marrow induced a protective memory against bacterial pathogen; whereas sub-cutaneous vaccination did not prime HSCs. Priming innate immune progenitor cells promoted myelopoiesis and the generation of trained monocytes/macrophages enhancing bacterial clearance. This was the first study exploring the mechanism of long term effects of BCG on trained immunity (Kaufmann et al., 2018).

Therefore, it is important to study the immunological memory capacity of stem cells beyond 1 month and their ability to transmit information to innate immune cells.

#### Hematopoietic Stem Cells

Kauffman and her colleagues published an important study which brings additional information to explain how cells transmit their immunological memory to progenitors, providing long-lasting protection (Kaufmann et al., 2018). These authors showed that in mice, the inoculation of bone marrow with BCG induced protection against *Mycobacterium tuberculosis* during a second infection which was not the case after subcutaneous inoculation. Specifically, it was shown that bone marrow BCG inoculation initially increased the amount hematopoietic stem cells (HSCs) and myelopoiesis and trained HSCs which offspring had the ability to develop a memory response against virulent *Mycobacterium tuberculosis*. Moreover, BCG induces epigenetic modifications of 2,483 genes in the macrophages, inducing changes of histone H3K27ac in (BMDM) Bone-marrow-derived macrophage produced from BCG-vaccinated mice compared with PBS-control mice (Kaufmann et al., 2018).

Another recent study showed that trained immunity performed at the level of bone marrow precisely increased myelopoeisis (Mitroulis et al., 2018). A total of 1,383 differentially expressed genes in β-glucan-injected mice compared to PBS-treated control mice, were involved in some innate immune functions such as production of pro-inflammatory cytokine IL-1β, production of GM-CSF (granulocyte-macrophage colony-stimulating factor) and immunometabolism including the biosynthesis of cholesterol and glycolysis (Mitroulis et al., 2018).

### Epithelial Stem Cells

Epithelial stem cells are progenitors of differentiated epithelial cells. Epithelial cells and fibroblasts are forming a physical and functional barrier against external agents (Sacco et al., 2004). They constitute the first line of defense in innate immunity (Ochiel et al., 2008). Like innate immune cell, epithelial cells can act as sentinel cells by expressing TLRs, producing immunomodulator factors and antimicrobial peptides when exposed to danger signals like bacteria, virus, and noxious signals (Schaefer et al., 2004; Bautista-Hernández et al., 2017). In addition to these immune characteristics, it was reported that skin epithelial stem cells exhibit trained immunity. By using an imiquimod (IMQ)-induced model of skin inflammation, the authors showed that the skin previously exposed to one inflammatory challenge responded faster to an unrelated secondary challenge, with faster wound healing in primed mice than in naïve mice (Naik et al., 2017). A recent review by Novakovic entitled “I Remember You: Epigenetic Priming in Epithelial Stem Cells” speculated that this memory of inflammation could be due to epigenetic mechanisms (Novakovic and Stunnenberg, 2017). Additionally, epithelial cell possess a long lifespan averaging 2 years (Tunn et al., 1989).

### Intestinal Stromal Cells

Intestinal stromal cells (iSCs) are part of non-professional immune cells including mesenchymal stem cells, fibroblasts, epithelial cells and endothelial cells that exhibit immune properties and contribute to immunity processing. ISC express TLRs 1–9, act as sentinel cells and produce pro-inflammatory cytokines in response to a pathogen (Owens and Simmons, 2013; Augenlicht et al., 2016). Recently, Owens assessed that iSCs possess the immune machinery to respond against bacterial pathogens and suggested that iSCs could exhibit an immunological memory (Owens, 2015). Indeed iSCs could develop a protective response to rapidly eliminate the pathogen or another microorganism during a second contact (Owens, 2015). ISCs produce prolonged pro-inflammatory cytokines (inflammatory memory) to recruit immune cells to the site of infection during a second contact (Owens and Simmons, 2013; Owens et al., 2013; Owens, 2015). Epigenetic modifications and immunometabolic mechanisms would be implicated in the persistence of immunological memory. Moreover, the lifespan of iSCs is longer than professional innate immune cells (Arts et al., 2016b; Hotamisligil, 2017; Smith et al., 2017).

### Fibroblasts

The main function of fibroblasts is to maintain the structural integrity of the connective tissue (Wong et al., 2007). Nevertheless, it has now been well established that fibroblasts play an important role in the immunity. Fibroblasts are equipped with 1 to 10 TLRs, produce pro-inflammatory and antimicrobial peptides, cytokines, chemokines and growth factors in response to pathogen invasion (Jordana et al., 1994; Van Linthout et al., 2014). Further, studies reported that fibroblasts are sentinel cells responding to pathogens and interacting with other cells via the production of molecular signals (Kaufman et al., 2001). Recent studies revealed that fibroblasts were involved in the persistence of inflammation. When activated with bacterial infection or danger signal (cytokines), tissue repair triggers a protective immune response by producing cytokines and interacting with immune cells (Flavell et al., 2008; Frank-Bertoncelj et al., 2017). The persistence of inflammation is controlled by epigenetic mechanisms. In response to injury or infection, fibroblasts first recruit immune cells in the site of infection to clear bacteria, then organize tissue repair and renewal (Flavell et al., 2008). Indeed, tissue repair and renewal are not random as fibroblasts possess a positional memory capacity enabling regenerating cells to recall spatial information from the uninjured tissue (Bustos-Arriaga et al., 2011; Van Linthout et al., 2014). This process is governed by epigenetic regulation of the homeobox “HOX” genes also involved in regulating body formation during development (Francis and Kingston, 2001; Coleman and Struhl, 2017). Much remains to be investigated in the immune functions of the fibroblasts such as the immunological memory. Functionally fibroblasts possess the immunological arsenal as mentioned above: the ability to produce inflammatory cytokines, antimicrobial peptides, and possess a longer lifespan than the cells of innate immunity such as macrophage once differentiated (Weissman-Shomer and Fry, 1975).

### Microglial Cells

Microglial cells are resident immune cells of the central nervous system (CNS) also known as resident macrophages of CNS (Wake and Fields, 2011). The main function of microglial cells is to ensure the homeostasis of synapses and the communication with the micro-environment in the CNS. Once activated by virus or bacteria, microglial cells somal size increases while retraction and thickening of processes are faciliting their migration capacity; they express TLRs, produce inflammatory cytokines and acquire phagocytic ability like macrophages (Mariani and Kielian, 2009). After this priming, microglial cells become susceptible to a secondary danger signal leading an improve immune response (Perry and Holmes, 2014; Haley et al., 2017). Several reviews suggested a process of trained immunity in microglial cells (Haley et al., 2017; Lelios and Greter, 2018; Wendeln et al., 2018). Haley recently speculated the fact that microglial cells possess an innate immune memory characterized by inflammatory pathways orchestrated with epigenetic mechanisms (Haley et al., 2017). A study comparing naive mice and mice primed with attenuated *Salmonella typhimurium* containing its LPS indicated that the second group exhibited increased microglial immunoreactivity in response to a second stimulation of LPS four weeks later. Interestingly, there was no increased immunoreactivy in microglial for the naive mice (Püntener et al., 2012). However, the authors did not investigate a potential epigenetic mechanism to explain the long-term activation of microglia in this infectious model.

## Conclusion

The established dogma that innate immunity system lacks memory and that only the adaptive immune system is able to recognize an infectious agent and to destroy it faster in a second encounter has been challenged. Current published data indicate that innate immune cells are able to build an immunological memory. Indeed, the new concept called ≪ trained immunity ≫ represents a paradigm change in the biology of immunity, emerging as a third way between the conventional dichotomy “innate immunity” and “adaptative immunity.” First report of trained immunity described an inflammatory protection of organism against a pathogenic microorganism upon a second encounter independent of adaptative immunity. It is initially concerned monocytes/macrophages and NK cells. However, in the last decade, it has been demonstrated that stem cells and other stromal cells display immune abilities, limited compared to professional innate immune cells, yet contributing to protective immune responses by humoral mediation (inflammatory). Eventually, trained immunity is also described in non-immune cells such hematopoetic stem cells and mesenchymal stem cells. The common mechanism of the non-immune cells in term of trained immunity is based in the ability to respond inflammatory stimuli. Like professional immune cells, a second challenge activates faster the production of cytokines. Consequently, this inflammatory ameliorates the immune response by rapid activation and recruitment of the immune cells at the site of infection, facilitating wound repair. Quantitative real time qRT-PCR array revealed that epigenetic modification mechanisms are involved in the instruction and establishment of inflammatory memory.

Finally, in order to avoid any confusion and to harmonize the concept of immunological memory, we propose to keep the term “trained immunity” because an increasing number of studies are showing that non-immune cells possess immunological memory which is therefore not restricted to innate immune cells.

## Author Contributions

AH and CT drafted the first version. EG and MD conceived the research and approved the final version.

### Conflict of Interest Statement

The authors declare that the research was conducted in the absence of any commercial or financial relationships that could be construed as a potential conflict of interest.
